# Learning Curve for Laparoscopic Repair of Pediatric Inguinal Hernia Using Percutaneous Internal Ring Suturing

**DOI:** 10.3390/children8040294

**Published:** 2021-04-11

**Authors:** Zenon Pogorelić, Dario Huskić, Tin Čohadžić, Miro Jukić, Tomislav Šušnjar

**Affiliations:** 1 Department of Pediatric Surgery, University Hospital of Split, 21000 Split, Croatia; tin.c94@gmail.com (T.Č.); mirojukic.mefst@gmail.com (M.J.); tomislav.susnjar@optinet.hr (T.Š.); 2 Department of Surgery, School of Medicine, University of Split, 21000 Split, Croatia; dariohuskic@gmail.com

**Keywords:** inguinal hernia, children, laparoscopy, percutaneous internal ring suturing, PIRS, learning curve

## Abstract

Background: Percutaneous internal ring suturing (PIRS) is a simple and popular technique for the treatment of inguinal hernia in children. The aim of this study was to analyze the learning curves during implementation of PIRS in our department. Methods: A total of 318 pediatric patients underwent hernia repair using the PIRS technique by three pediatric surgeons with different levels of experience in laparoscopic surgery. These patients were enrolled in a prospective cohort study during the period October 2015–January 2021. Surgical times, intraoperative and postoperative complications, in addition to outcomes of treatment were compared among the pediatric surgeons. Results: Regarding operative time a significant difference among the surgeons was found. Operative time significantly decreased after 25–30 procedures per surgeon. The surgeon with advanced experience in laparoscopic surgery had significantly less operative times for both unilateral (12 (interquartile range, IQR 10.5, 16.5) min vs. 21 (IQR 16.5, 28) min and 25 (IQR 21.5, 30) min; *p* = 0.002) and bilateral (19 (IQR 14, 21) min vs. 28 (IQR 25, 33) min and 31 (IQR 24, 36) min; *p* = 0.0001) hernia repair, compared to the other two surgeons. Perioperative complications, conversion, and ipsilateral recurrence rates were higher at the beginning, reaching the benchmarks when each surgeon performed at least 30 PIRS procedures. The most experienced surgeon had the lowest number of complications (1.4%) and needed a fewer number of cases to reach the plateau. The other two surgeons with less experience in laparoscopic surgery had higher rates of complications (4.4% and 5.4%) and needed a higher number of cases to reach the plateau (*p* = 0.190). Conclusions: A PIRS learning curve for perioperative and postoperative complications, recurrences, and conversion rates reached the plateau after each surgeon had performed at least 30 cases. After that number of cases PIRS is a safe and effective approach for pediatric hernia repair. A surgeon with an advanced level of experience in pediatric laparoscopic surgery adopted the technique more easily and had a significantly faster learning curve.

## 1. Introduction

After the introduction of percutaneous internal ring suturing (PIRS) for pediatric inguinal hernia repair by Patkowski, this technique is now performed routinely in a number of pediatric surgery centers worldwide [1,2]. In this technique only a single 3 mm umbilical port is used for achieving pneumoperitoneum and for the introduction of a laparoscope. PIRS involves the percutaneous closure of the internal inguinal ring using a spinal needle under the control of a laparoscope [1,2]. If compared to conventional three port laparoscopic pediatric hernia repair, this surgical technique is technically easier with a shorter learning curve, since there is no intracorporeal suturing [1,2,3,4]. Several benefits have been reported including shorter operative time, better cosmetics, no need for tracheal intubation, lower recurrence rates and less possibility of complications [3,4,5,6,7]. A significantly lower level of pain and inflammatory stress response has been reported using the PIRS technique [4,8]. 

After the introduction of the PIRS technique first published reports were associated with higher recurrence rates and residual hydroceles [1]. New studies have reported recurrence rates of 0.5–0.7%, which is very similar to those in open surgery [2,4]. Additionally, operative time was reported to be lower than open surgery, with unilateral hernias averaging 10 min and bilateral hernias 15 min [9,10]. Incidence of complications in the hands of experienced laparoscopic pediatric surgeon is very low. The most common reported complications are injury to blood vessels, recurrence, hydrocele formation, knot reaction, severe pain, and scrotal swelling [1,4,9,10]. Care should be taken not to injure the spermatic cord or vessels, although the risk is significantly higher in traditional open surgery [1,9,10]. In the case of vascular structure injuries, laparoscopic surgery may be completed only in the absence of further bleeding and growth of retroperitoneal hematoma. If that is not possible, conversion to open surgery should be performed [10].

Another important issue with regard to laparoscopic surgery is the existence of a learning curve. As known from many laparoscopic procedures, a number of are required before the technique is safely performed. In general, learning curves compare the relationship between experience and outcome. The first use of these curves in medicine came in the 1980s to describe the attainments of new surgical skills in minimally invasive surgeries such as laparoscopy [11]. Nowadays, the use of these curves is steadily increasing in research, randomized control trial design, competency assessment, healthcare education, and for the design of various training programs [11]. Learning curves in surgery are related to training and the attainment of medical education. When a surgeon learns a new procedure they generally improve with experience, and plotting experience versus performance as a graph results in the creation of a learning curve [12]. The outcomes related to learning a new surgical method can be measured as surgical process and patients’ outcomes. Factors involved in the surgical process can include operative length, blood loss, re-operation rate, conversion to open procedure in minimally invasive surgeries, extent of resection, margin involvements and lymph node yield [12]. Learning curves are complex functions and as a result contain various stages. The beginning stage is known as the initial curve, where there is generally a stepwise improvement in learning that can be applied across all medical specialties and procedures. There is a theory that states that learning occurs slower when an operator becomes more competent at a skill. This inevitably leads to reaching an expert plateau [12]. Some factors that may affect the rate of learning are related to the individual at hand, such as previous experience, motivation, natural talent and the ability to acquire new skills. The plateau that is inevitably reached does not necessarily indicate an expert level but instead signifies when retardation of learning has occurred. The last stage is the redirection of performance. After the plateau has been reached there is usually a slight decline in performance, which can usually be attributed to overconfidence and the ascertainment of more difficult operations [11]. A recent study reported that there is a significant reduction in the number of intraoperative complications and recurrence rates after 35 PIRS procedures per pediatric surgeon [13]. Another study showed that although there were individual differences, all trainees acquired the skill to perform PIRS adequately within ten months [14]. 

The aim of this study is to investigate how much experience is needed to acquire the skill to perform PIRS safely. The secondary aim is to determine if previous experience in laparoscopic surgery might shorten the learning curve.

## 2. Materials and Methods

### 2.1. Patients

A total of 318 pediatric patients who underwent hernia repair using the PIRS technique in the period from October 2015 to January 2021 were enrolled in this prospective cohort study. Four patients were excluded from the study because they met at least one exclusion criterion. Ultimately, 314 patients were included in the study. Inclusion criteria were patients aged 0 to 17 years of age, who underwent PIRS for inguinal hernia by one of three pediatric surgeons included in the study, and were followed up for more than three months after surgery. Exclusion criteria were patients older than 17 years of age, and patients lost from follow-up or that have been followed up for less than three months. Parents or legal representatives of the children signed informed consent. The study protocol was approved by the Institutional Review Board of our hospital (reference 2181-147-01/06/M.S.-20-9). For each patient, medical history, demographic data, lateralization of hernia, intraoperative findings, surgical time, level of pneumoperitoneum, duration of hospitalization, intraoperative or postoperative complications, outcomes of treatment and recurrences were recorded. All surgery was performed by three pediatric surgeons with different level of experience in laparoscopy. At the beginning of the study the first surgeon (Surgeon A) was 28 years of age and had basic experience in laparoscopic surgery, the second surgeon (Surgeon B) was 35 years of age and had advanced experience in laparoscopic surgery while the third surgeon (Surgeon C) was 53 years of age and had moderate experience in laparoscopic surgery.

### 2.2. Outcome Measurements

The primary outcome of the study was to evaluate learning curve in regards to operation time and complications. The secondary outcome was to determine whether the age of the surgeon and previous experience in laparoscopic surgery influenced the learning curve.

### 2.3. Surgery

**Figure 1:**
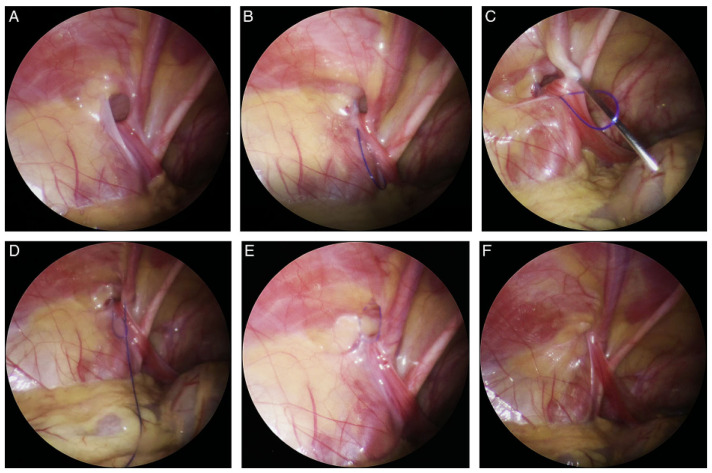
PIRS technique: (**A**)—Open internal inguinal ring; (**B**)—Introduction of a nylon loop; (**C**)—Introduction of the needle on the other side of the internal ring; (**D**)—Needle and suture passed through the previously introduced loop; (**E**)—The loop drawn out and the knot passed around internal ring; (**F**)—Closed internal ring.

A Veress needle was used to achieve pneumo-peritoneum (6–10 mm, depending on patient’s age and weight). After achieving pneumo-peritoneum, a 3-mm incision in supraumbilical region was performed. The abdominal cavity was inspected using a 3-mm laparoscope (Karl Storz, Tuttlingen, Germany). After visualization of an open internal inguinal (Figure 1A) ring, a mini skin incision was performed above the internal ring on the side of the hernia. A non-absorbable monofilament nylon loop was introduced sub-peritoneally at one side of the internal ring using a 20G spinal needle (Figure 1B). After the loop was successfully introduced, the spinal needle was taken out. On the other side of the internal ring through the same skin incision, the same needle was introduced again but now passing through the previously introduced loop for introduction of the nylon suture (Prolene™ 3-0, polypropylene, Ethicon^®^, Cincinnati, Ohio, USA) (Figure 1C). After introducing enough length of the suture, the needle was removed again, the loop was withdrawn (Figure 1D), and the suture was taken and passed out of the skin incision. Thereafter the suture was placed around the entire circumference of the internal ring opening (Figure 1E). The suture was tied and the internal ring was completely obliterated (Figure 1F). Incisions were closed using braided adhesive skin closures (3M^TM^ Steri-Strip^TM^, Neuss, Germany).

### 2.4. Follow-Up

Intraoperative (injury of blood vessels) and postoperative complications (hydrocele, swelling in the groin, recurrence) were recorded in the study protocol. Most of the cases (88%) were performed as day cases. The patients were followed up at our outpatient clinic. Braided adhesive skin closures were removed after seven days at first visit. Follow-up program consisted of physical examination after one, six and twelve months to assess the presence of late complications or recurrence of the hernia. 

### 2.5. Statistical Analysis

SPSS 24.0 software (IBM Corp, Armonk, NY, USA) was used to analyze the data. Median and interquartile range (IQR) were used for analysis of the quantitative variables or for the ordinal variables. To describe a distribution of categorical variables, absolute and relative frequencies were used. The significance of differences in quantitative variables between the surgeons was assessed by the analysis of variance (ANOVA). The chi-square test was used to assess differences in distribution of categorical data. When the frequency of events in a certain cell was low, Fisher exact test was used instead. All the tests were two-sided and a significance level of 0.05 was used. 

## 3. Results

A total number of 372 PIRS procedures in 314 children (126 (67%) males) with median age of 4.5 years (IQR 2, 6.5) were performed. There were 181 (57.6%) right, 75 (23.9%) left, and 58 (18.5%) bilateral hernia repairs. Among all performed PIRS procedures there were three conversions to open surgery. The median duration of surgery (time between first incision to completion of the skin closure) for unilateral and bilateral inguinal hernias was 16.5 min (IQR 15, 25) and 21.2 min (IQR 20.8, 23.8), respectively. The median postoperative hospital stay was one (IQR 1, 1) day and median follow-up was 44 (IQR 32, 50) months. During the study period there were four (1.3%) intraoperative complications and seven (2.2%) postoperative complications. Regarding intraoperative complications four (1.3%) inferior epigastric vein injuries were recorded, and injuries were treated conservatively without any consequences. During the follow-up period in five male children hydrocele was recorded, in four cases hydrocele resolved spontaneously within three months, while one (0.3%) required surgical treatment. In two (0.6%) patients swelling of the tissues around the upper pole of the groin was recorded, which finally resolved up to six months after surgery. One case (0.3%) of hernia recurrence was recorded.

### 3.1. Individual Learning Curve

Three surgeons in our department adopted PIRS as the technique of choice for treatment of pediatric inguinal hernia. Each of these performed a minimum of 74 surgeries. Demographic characteristics of the patients were similar among the surgeons (Table 1). Regarding operative time, significant differences among the surgeons was found. Operative time decreased significantly after 25–30 procedures per surgeon.

**Figure 2:**
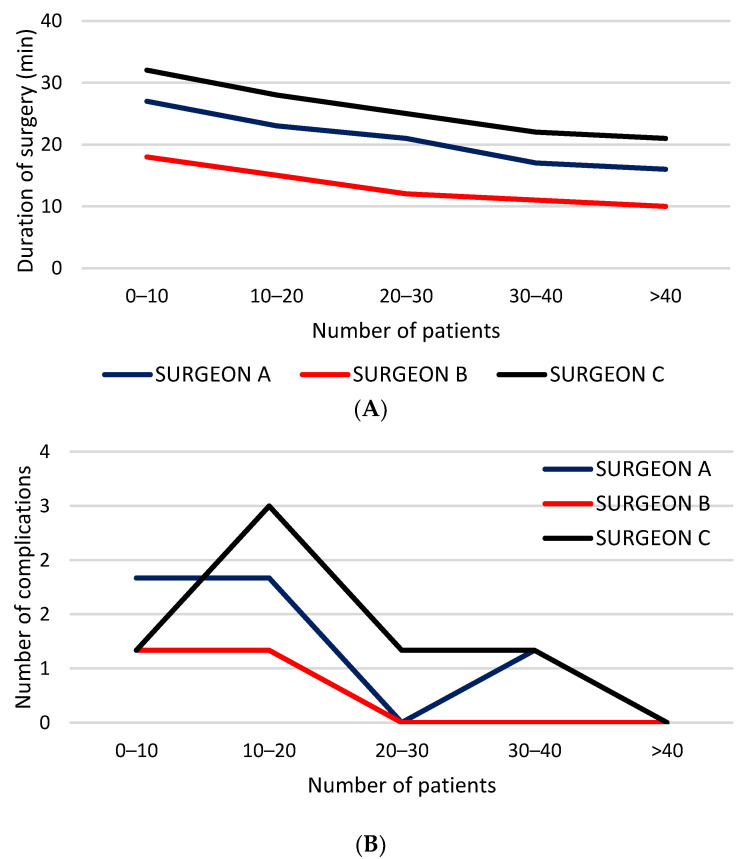
Individual learning curve: (**A**) duration of surgery; (**B**) complication rates.

The surgeon with advanced experience in laparoscopic surgery had significantly less operative times for both unilateral (*p* = 0.002) and bilateral (*p* = 0.0001) hernia repair, compared to the other two surgeons (Figure 2A). A decline of intraoperative complications was observed along with accumulated individual experience (Figure 2B).

Blood vessel injuries reached their nadir at the 20th surgery. No hydrocele and recurrence occurred after each surgeon’s 33rd and 22nd case, respectively (Table 2). There were no conversions after the 17th case of each surgeon. Individual differences between the surgeons regarding complications was also seen. The most experienced surgeon had the lowest number of complications (1.4%) and needed a fewer number of cases to reach the plateau while the other two surgeons with less experience in laparoscopic surgery had higher rates of complication (4.4% and 5.4%) and needed a higher number of cases to reach the plateau, but this difference was not statistically significant (*p* = 0.190). Similar findings were found regarding conversion rates. The most experienced surgeon had a conversion rate of 0% while the other two surgeons had conversion rates of 1.1% and 2.7%, respectively (*p* = 0.432).

### 3.2. Department Learning Curve

**Figure 3:**
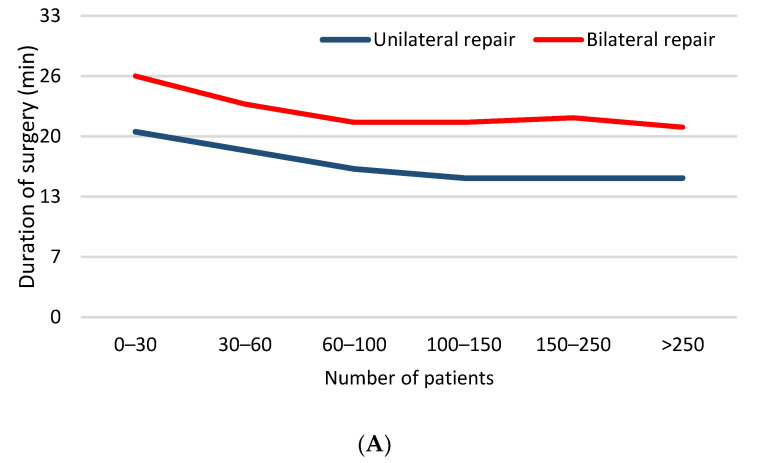

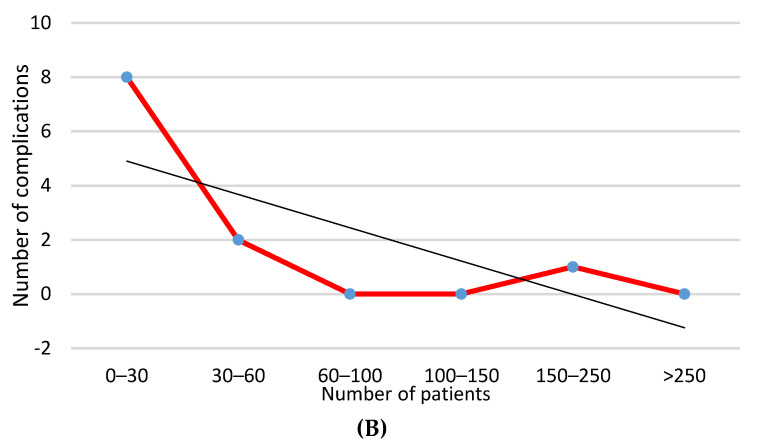
Department learning curve: (**A**) duration of surgery; (**B**) complications–blood vessels injury, hydrocele formation, swelling in the groin.

In the department-centered analysis, operative time for both unilateral and bilateral repair was significantly higher at the beginning of the study and achieved plateau after 45–60 patients (*p* < 0.001) (Figure 3A). Complication rates were significantly greater in the first 30 patients and after that the number of complications significantly decreased (Figure 3B). Conversion and recurrence rates decreased to zero after the first 30 patients (Table 3).

## 4. Discussion

This study assessed learning curves of three surgeons from the same department with different level of experience in pediatric minimally invasive surgery. The selected procedure was PIRS for treatment of indirect inguinal hernia in the pediatric population. This study provided clear evidence that the duration of surgery was significantly reduced after 60–75 operated children independent of operating surgeon, or 25–30 subjects per operating surgeon. The overall number of intraoperative and postoperative complications also significantly decreased after 30–45 operated patients (independent on operating surgeon), and reached a minimum after 60 procedures. After 25–30 procedures per surgeon, the number of complications decreases to a minimum. The most common intraoperative complication was puncture of epigastric veins and the most common postoperative complication was hydrocele formation, most of which resolved spontaneously. Conversion rate was low and there were no conversions after the 17th case of each surgeon. The surgeon with advanced experience in laparoscopic surgery had a significantly fewer number of complications and shorter operative times compared to the other two surgeons. Generally, 30 PIRS procedures per surgeon are required for perioperative complications, conversion rate and ipsilateral recurrence to reach the benchmark. For surgeons with advanced experience in pediatric minimally invasive surgery. 15–20 procedures per surgeon are enough to reach the benchmark. The drawback in operative time was evident after 20 procedures per surgeon and reaches plateau after 30 procedures.

Open repair through inguinal incision which includes high ligation and resection of the hernia sac has been standard treatment for pediatric hernia for a long period of time [15]. With the development of minimally invasive surgery numerous laparoscopic techniques for pediatric inguinal hernia repair have been developed including intracorporeal suturing and an extracorporeal approach [1,2,3,4,14,16,17]. According to recent literature PIRS for pediatric inguinal hernia repair has been proven to be a safe and effective method [1,2,3,4,6,10]. It still remains an open question as to how much experience a surgeon needs to have to be able to perform this method safely without significant complications. Controversy remains regarding whether it is adequate for pediatric surgeons or trainees with a low level of experience to perform the procedure safely. Shibuya et al. concluded that, although there were individual differences, all trainees acquired the skill to perform PIRS adequately within ten months [14]. Similar findings were seen in our study. One resident of pediatric surgery was included in this study. He showed significant improvement after the first 25–30 cases, and after reaching plateau his results were better than the results of the older surgeon with moderate experience in laparoscopic surgery. 

Traditional laparoscopic hernia repair is time-consuming due to intracorporeal suturing and placement of multiple trocars. It has also been associated with increased postoperative pain and a higher recurrence rate [6,18]. PIRS has been associated with a decreased level of pain due to decreased surgical stress and inflammatory response [4,8]. Medians of surgical times for unilateral and bilateral repairs using PIRS technique are 11 to 19 min and 18 to 24 min, respectively [2,3,4,5]. This is the most substantial report describing the individual learning curve of PIRS by evaluating the operative time. Although operative time does not reflect the operative skill as it is, smoothly performing the procedure is essential for the safety of the surgery. In this study operative time decreased significantly after 25–30 procedures per surgeon, and the surgeon with advanced experience in laparoscopic surgery had significantly fewer operative times. Similar findings were reported in the literature [13,14].

In the pediatric population the incidence of recurrence rates following open inguinal hernia repair is 0.5–4% [11,15]. Recurrence rates following standard three port laparoscopic hernia repair is slightly higher and ranges between 0.7–4.5% [16,17,18,19,20,21]. After introduction of PIRS technique the first published reports had been associated with higher recurrence rates and residual hydroceles [4,13]. This had been influenced by the inexperience of the surgeons, the use of absorbable suture, and the use of a single suture or larger defects [21]. Recently, it has been described that PIRS resulted in a significant reduction of recurrence rates and operative time [2,3,4,5,10]. In this study postoperative hydrocele formation and recurrence rates were significantly greater in the first 30 patients and after that number of complications significantly decreased. Barroso et al. reported similar findings in their study [13]. Many pediatric surgeons selected female patients to start with as their anatomy appears more favorable [3]. Barroso et al. reported that most recurrences, in procedures performed by less experienced surgeons, occurred in females, because in many of them there is a fold of peritoneum under the round ligament that might easily be overlooked [13]. We agree with this observation. Our most experienced surgeon supervised many of the cases of less experienced surgeons and found a similar situation a few times during the surgery, when a less experienced surgeon overlooked a fold of peritoneum. That was corrected during the surgery without any consequences. Our only recurrence was in a female patient.

The most common intraoperative complication is injury of epigastric or iliac blood vessels [1,2,5,10,14]. Special attention is required while introducing the needle or manipulating the needle around the internal inguinal ring in order to avoid injury to surrounding blood vessels [10]. In this study epigastric vein puncture was the most common intraoperative complication which reached its plateau at the 20th surgery. The more experienced surgeon had a lower incidence of this complication.

This study demonstrates that, independent of previous surgical experience in minimally invasive surgery, pediatric surgeons easily adhere to the implementation of a minimally invasive program to repair inguinal hernia. However, limitations of this study are a relatively small population size and short follow-up period. Results of this study would need to be correlated with further analysis based on more pediatric PIRS cases.

## 5. Conclusions

A PIRS learning curve for perioperative and postoperative complications, recurrences, and conversion rates reached the plateau after each surgeon performed at least 30 cases. After that number of cases PIRS is a safe and effective approach for pediatric hernia repair. Surgeons with an advanced level of experience in pediatric laparoscopic surgery adopted the technique more easily and had significantly shorter operating times as well as a lower number of perioperative and postoperative complications.

## Figures and Tables

**Table 1:** Comparison of demographic data and treatment outcomes among surgeons.

		Surgeon A	Surgeon B	Surgeon C	*p*
		(*n* = 90)	(*n* = 150)	(*n* = 74)	
Demographic data of the patients					
Age (years)		5	4.5	4.5	0.571 *
Median (IQR)		(2, 6.5)	(2, 6)	(1.5, 7)	
Gender	Male	62 (69)	101 (67)	46 (62)	0.636 ^†^
n (%)	Femele	28 (31)	49 (33)	28 (38)	
Weight (cm)		111	109	109	0.441 *
Median (IQR)		(92, 123)	(94, 130)	(97, 128)	
Height (kg)		18	18	18.5	0.801 *
Median (IQR)		(14, 25)	(14, 26.5)	(13, 26)	
BMI (kg/m^2^);		17	18	17.5	0.684 *
Median (IQR)		(14, 17.7)	(14.5, 18.1)	(14.9, 18.6)	
ASA classification, *n* (%)					
	ASA I	84 (93)	139 (93)	68 (92)	0.891 ^†^
	ASA II	6 (7)	11 (7)	6 (8)	
Lateralization, *n* (%)					
	Left	21 (23.5)	35 (23)	19 (25.5)	
	Right	49 (54.5)	90 (60)	42 (57)	0.839 ^†^
	Bilateral	20 (22)	25 (17)	13 (17.5)	
Outcomes of treatment					
Duration of surgery (min)					
	Unilateral repair	21 (16.5, 25)	12 (10.5, 16.5)	25 (21.5, 30)	0.002 *
	Bilateral repair	28 (25, 33)	19 (14, 21)	31 (24, 36)	0.0001 *
Hospital stay (days)		1	1	1	>0.999 *
Median (IQR)		(1, 1)	(1, 1)	(1, 1)	
Conversions, *n* (%)		1 (1.1)	0	2 (2.7)	0.432 ^‡^
Complications, *n* (%)		4 (4.4)	2 (1.4)	4 (5.4)	0.190 ^‡^
	Hydrocele	2 (2.2)	1 (0.7)	2 (2.7)	
	Blood vesels injury	2 (2.2)	1 (0.7)	1 (1.4)	
	Swelling in the groin	1 (1.1)	0	1 (1.1)	
	Recurrence	0	0	1 (1.4)	

* ANOVA; ^†^ Chi square test; ^‡^ Fisher exact test; IQR—Interquartile range; BMI—Body mass index; ASA—American Society of Anesthesiologists.

**Table 2:** Individual learning curve—outcomes of the treatment.

	Number of the Patients				
	0–10	10–20	20–30	30–40	>40
Duration of surgery–unilateral repair (min)					
SURGEON A	27	23	21	17	16
SURGEON B	18	15	12	11	10
SURGEON C	32	28	25	22	21
Blood vessels injury (*n*)					
SURGEON A	1	1	0	0	0
SURGEON B	1	0	0	0	0
SURGEON C	0	1	0	0	0
Complications–Hydrocele/Recurrence (*n*)					
SURGEON A	0	1	0	1	0
SURGEON B	0	1	0	0	0
SURGEON C	0	1	1	1	0
Conversion rate (*n*)					
SURGEON A	1	0	0	0	0
SURGEON B	0	0	0	0	0
SURGEON C	1	1	0	0	0

**Table 3:** Department learning curve—outcomes of the treatment.

	Number of the Patients					
Outcome	0–30	30–60	60–100	100–150	150–250	>250
Duration of surgery (min)						
Unilateral repair	20	18	16	15	15	15
Bilateral repair	26	23	21	21	21.5	20.5
Complications (*n*)						
Blood vessels injury	4	0	0	0	0	0
Hydrocele	4	1	0	0	0	0
Swelling in the groin	1	0	0	0	1	0
Recurrence (*n*)	1	0	0	0	0	0
Conversion rate (*n*)	2	1	0	0	0	0

## Data Availability

The data presented in this study is available upon request of the respective author. Due to the protection of personal data, the data is not publicly available.
